# Transcriptome profiling reveals the effects of drought tolerance in Giant Juncao

**DOI:** 10.1186/s12870-020-02785-7

**Published:** 2021-01-04

**Authors:** Jing Zhou, Siqi Chen, Wenjiao Shi, Rakefet David-Schwartz, Sutao Li, Fulin Yang, Zhanxi Lin

**Affiliations:** 1 National Engineering Research Center of Juncao, Fujian Agriculture and Forestry University, Fuzhou, 350002 China; 2 College of Animal Sciences (College of Bee Science), Fujian Agriculture and Forestry University, Fuzhou, 350002 China; 3 Institute of Plant Sciences, Volcani Center, Agriculture Research Organization, 50250 Bet Dagan, Israel

**Keywords:** Giant Juncao, RNA-sequence, Transcriptome, Drought resistance, Transcription factors

## Abstract

**Background:**

Giant Juncao is often used as feed for livestock because of its huge biomass. However, drought stress reduces forage production by affecting the normal growth and development of plants. Therefore, investigating the molecular mechanisms of drought tolerance will provide important information for the improvement of drought tolerance in this grass.

**Results:**

A total of 144.96 Gb of clean data was generated and assembled into 144,806 transcripts and 93,907 unigenes. After 7 and 14 days of drought stress, a total of 16,726 and 46,492 differentially expressed genes (DEGs) were observed, respectively. Compared with normal irrigation, 16,247, 23,503, and 11,598 DEGs were observed in 1, 5, and 9 days following rehydration, respectively. Gene Ontology and Kyoto Encyclopedia of Genes and Genomes pathway analyses revealed abiotic stress-responsive genes and pathways related to catalytic activity, methyltransferase activity, transferase activity, and superoxide metabolic process. We also identified transcription factors belonging to several families, including basic helix-loop-helix (bHLH), WRKY, NAM (no apical meristem), ATAF1/2 and CUC2 (cup-shaped cotyledon) (NAC), fatty acyl-CoA reductase (FAR1), B3, myeloblastosis (MYB)-related, and basic leucine zipper (bZIP) families, which are important drought-rehydration-responsive proteins. Weighted gene co-expression network analysis was also used to analyze the RNA-seq data to predict the interrelationship between genes. Twenty modules were obtained, and four of these modules may be involved in photosynthesis and plant hormone signal transduction that respond to drought and rehydration conditions.

**Conclusions:**

Our research is the first to provide a more comprehensive understanding of DEGs involved in drought stress at the transcriptome level in Giant Juncao with different drought and recovery conditions. These results may reveal insights into the molecular mechanisms of drought tolerance in Giant Juncao and provide diverse genetic resources involved in drought tolerance research.

**Supplementary Information:**

The online version contains supplementary material available at 10.1186/s12870-020-02785-7.

## Background

Drought stress is one of the most threatening environmental constraints that adversely affect plant growth and yield [1]. However, with global climate change, the frequency and intensity of drought have continuously increased [2, 3]. Drought might cause metabolic imbalance in plant cells and influence the optical energy absorption of plant leaves, destroying the photosynthetic organs of plants [4]. Moreover, drought can lead to the accumulation of active oxygen substances in the leaves, which may accelerate the peroxidation of biological membrane lipid to produce toxic products, thereby inhibiting plant growth [5]. For plants exposed to drought stress, the total primary productivity is not only closely related to their resistance and tolerance to drought stress, but also shows an important relationship with the ability of plants to recover from damage after the elimination of the stress [6, 7]. Therefore, the recovery ability after rehydration is important for the successful adaptation of plants to arid environments. Rehydration helps plants recover their physiological functions, and it can offset plant damage from drought stress to a certain extent [7]. However, the compensation of rehydration to plant growth after drought stress is often limited. The recovery degree of plant growth might be related to the degree and duration of drought stress before rehydration and drought resistance of plants [8].

To cope with drought stress, plants have adapted various self-protection and defense mechanisms in the long-term evolution process [9]. Plants decrease the photosynthetic capacity of mesophyll by closing the stomata to adjust the photosynthetic process of leaves [8]. By adjusting the in vivo antioxidation system, plants can eliminate excess active oxygen and maintain in vivo redox equilibrium. The intracellular water potential can be increased by increasing the substances of cell osmotic adjustment to maintain a certain expansion, thereby protecting the continuous growth of plants under drought stress [10]. Plant hormones regulate their own response mechanism through synergistic or antagonistic action in response to arid states. Various expressed genes have been reported in response to drought stress [11]. These genes include stabilizing membrane proteins, heat shock proteins and late embryogenesis abundant proteins, which play an important role in stabilizing protein structure and enhancing cell’s water binding capacity. Early drought-induced proteins protect plants by producing certain metabolic proteins and regulating gene expression through precise signal transduction during drought stress. Dehydrin genes (*Dhn*), which are among the most frequently observed proteins in plants, protect the cells from water deficit. Additionally, a large number of genes change their expression through the regulation of transcription factors (TFs) [12]. Several TFs also provide response under drought stress, including abscisic acid-responsive element (ABREs) binding factors (ABFs, AREBs, or DPBFs) [13–15], dehydration-responsive element binding factors [16], myeloblastosis (MYB), and SNF1-related kinase 2 [17]. Therefore, knowledge about the various genes translated and expressed in response to drought stress conditions will help elucidate the water deficit tolerance mechanisms and facilitate the development of new plant cultivation tools to combat climate change.

Giant Juncao is an ideal Gramineae C_4_ plant for water-soil conservation, wind prevention, and sand fixation due to its large biomass, disease resistance, and developed root system. This plant has been widely applied for the comprehensive environmental management of regions with vulnerable ecology [18]. However, drought environment influences the yield and restricts its large-scale plantation. To improve its productivity and performance in water deficit conditions, the response mechanisms of Giant Juncao to drought should be elucidated. Recently, the development of molecular biological methods has facilitated the discovery of potential plant response mechanisms to environmental stresses. RNA sequencing (RNA-seq) can quickly and comprehensively obtain the gene expression of a specific cell or tissue in a certain state, so as to determine the molecular mechanism of physiological metabolic responses of plants under abiotic stress conditions [19]. RNA-seq data could provide insights into the discovery of new genes, including annotation genes and differentially expressed genes (DEGs), and molecular markers [20]. Compared with traditional sequencing methods, RNA-seq provides high-throughput sequencing results, is inexpensive, has high sensitivity, and can detect low abundance expressed genes [21]. A large quantity of DEGs associated with drought stress response have been reported in various Gramineae species by RNA-seq, such as wheat [22], maize [23], rice [24], sorghum [25], and foxtail millet [26]. This method can obtain transcripts from different developmental stages, tissues, and organs, so it is a fundamental and efficient method for discovering functional genes.

Herein, we aimed to identify the genes involved in the response of Giant Juncao to drought stress and rehydration treatment using Illumina sequencing. We analyzed DEGs in Giant Juncao seedlings to elucidate its molecular response mechanism to drought stress. Furthermore, we employed the physiological indices to understand the drought response mechanism of Giant Juncao. As only a few studies have identified genes that respond to drought stress in *Pennisetum* spp., our study provides an important transcriptomic database for further targeted gene modifications in grasses.

## Results

### Physiological evaluation of Giant Juncao seedling in response to drought and rehydration treatment

**Fig. 1:**
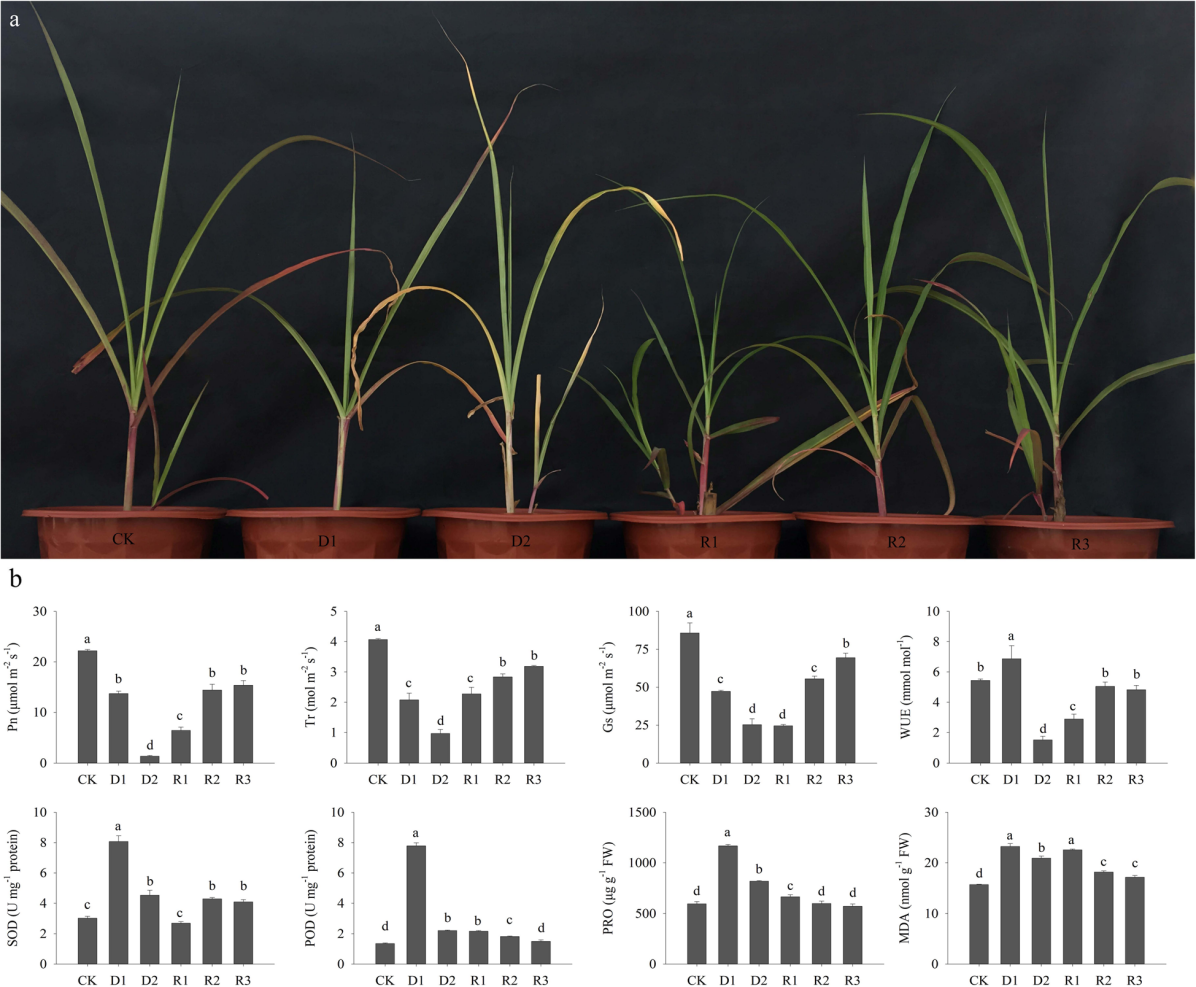
Effect of drought stress and rehydration condition on the growth of Giant Juncao. **a** A picture of Giant Juncao under different treatments condition. **b** physiological and ecological indicators between drought stress and rehydration treatment of Giant Juncao. Different lowercased letters in the same panel indicate statistical significance (*P* < 0.05). Pn: net photosynthetic rate, Tr: transpiration rate, Gs: stomatal conductance, WUE: water use efficiency, SOD: superoxide dismutase, POD: peroxidase, PRO: proline, MDA: malondialdehyde; *P <* 0.05

Drought stress and rehydration treatments affected the physiological index of Giant Juncao (Fig. 1a). Compared with the control, drought-stressed plants showed a significant decrease in Pn, Tr, and Gs at D1 and D2, whereas the WUE increased at D1 and decreased at D2 (Fig. 1b). After rehydration, all ecological indices except Gs significantly increased compared with those after 14 days of drought stress. With the increase in recovery time, the ecological index of Giant Juncao reached a stable stage during 5 and 9 days of rehydration, and the Gs steadily increased. The Pn was 14.43–15.40 μmol m^− 2^ s^− 1^ for nearly 7 days of drought stress. The Tr and Gs were 2.84–3.19 mol m^− 2^ s^− 1^ and 55.50–69.50 μmol m^− 2^ s^− 1^, respectively, which were higher than those in plants exposed to 7 days of water deficit. The WUE increased to 5.06 mmol mol^− 1^, which showed no significant difference with the control when the plant was re-watered for 5 days (Fig. 1b).

After drought stress, SOD, POD, PRO, and MDA showed an increasing and decreasing trend. Since the re-watering, the SOD and PRO rapidly decreased (Fig. 1b). The POD remained stable, and MDA significantly increased when watering was restored for 1 day. With the increasing rehydration time, POD, PRO, and MDA exhibited a continued declining trend, but SOD showed the opposite tendency. At the end of the experiment, SOD and MDA levels were 4.09 U mg^− 1^ protein and 17.16 nmol g^− 1^ FW, respectively, which were significantly higher than those under non drought stress (*P* < 0.05), whereas the POD and PRO were similar to those in the control.

### Transcriptome sequencing and assembly

In this study, 18 cDNA libraries from the non drought stress (0 day), drought stress (7 and 14 days), and rehydration (1, 5, and 9 days) conditions were constructed totally, and then C0, D1, D2, R1, R2, and R3 were referred correspondingly. Each condition had three biological repetitions. An overview of the sequencing is listed in Table S1, and more than 97.18% and approximately 94% of the bases from the over 1 billion raw reads had a *q*-value ≥20 and ≥ 30 (an error probability of 0.02–0.025%), respectively. The GC-content was between 55.67 and 57.76% (Table S1). All original FASTQ data files were submitted to the NCBI Sequence Read Archive (SRA), under accession numbers is PRJNA632455.

After filtering out low-quality reads, a total of 967 million clean reads were generated from six samples (Table S1). Trinity was used to generate 144,806 transcripts with N50 of 1705 bp and N90 of 645 bp (Table 1). Among them, 93,907 were unigenes, where less than 300 bp has 3630 unigenes, 300–500 bp has 16,003 unigenes, 500–1000 bp has 35,098 unigenes, 1–2 kb has 25,770 unigenes, and > 2 kb has 13,406 unigenes.

**Table 1:** Summary statistics of Giant Giant Juncao transcriptome assemblies

Nucleotides Length (bp)	Transcripts	Unigenes
<300	4033	3630
300–500	19,873	16,003
500–1000	49,966	35,098
1000–2000	45,423	25,770
>2000	25,511	13,406
Total	144,806	93,907
Shortest length	201 bp	201 bp
Median length	1291 bp	1163 bp
Longest length	14,660 bp	14,660 bp
N50	1705 bp	1541 bp
N90	645 bp	574 bp

### Functional and pathway annotation of genes

**Fig. 2:**
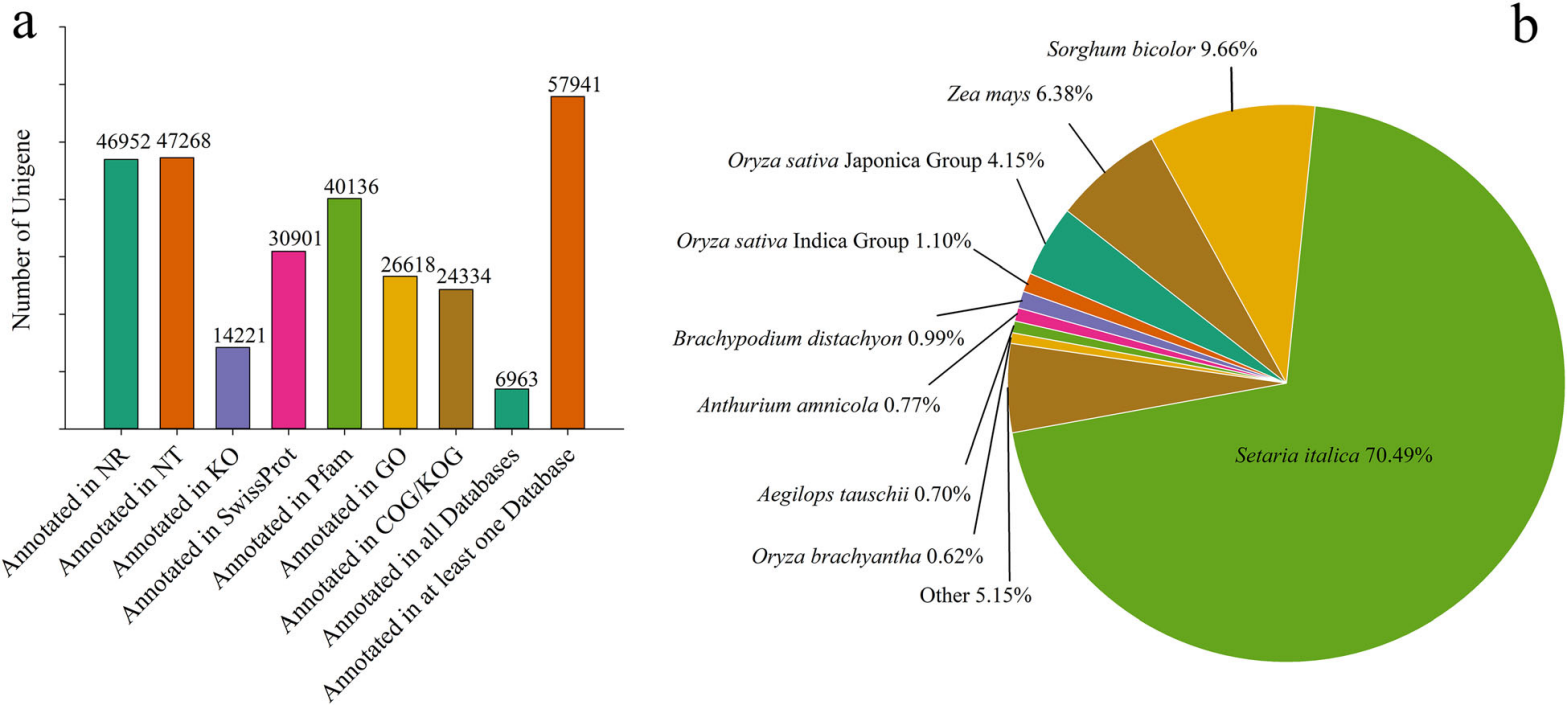
Information of unigenes annotated in different databases (**a**) and distributed into different species (**b**)

In order to investigate the genes’ function, all the assembled unigenes were annotated into the Nr, Nt, Swiss-Prot, KOG, Pfam, GO, and KEGG pathway databases (see Materials and Methods). A total of 57,941 unigenes were annotated in at least one database, which account for 61.7% of the unigenes (Fig. 2a, Table S2). A total of 6963 (7.4%) unigenes were annotated in the public databases (Fig. 2a). The numbers of unigenes in Nr, Nt, KEGG, Swiss-Prot, Pfam, GO, and KOG databases were 46,952 (50.0%), 47,268 (50.3%), 14,221 (15.1%), 30,901 (32.9%), 40,136 (42.7%), 26,618 (28.4%), and 24,334 (25.9%), respectively (Fig. 2a, Table S2). The unigenes matched sequences from the genomes of *Setaria italica*, *Sorghum bicolor*, *Zea mays*, *Oryza sativa Japonica* Group, *Oryza sativa Indica* Group, *Brachypodium distachyon*, *Anthurium amnicola*, *Aegilops tauschii*, *Oryza brachyantha*, and others (Fig. 2b).

### DEGs under drought and rehydration conditions

**Fig. 3:**
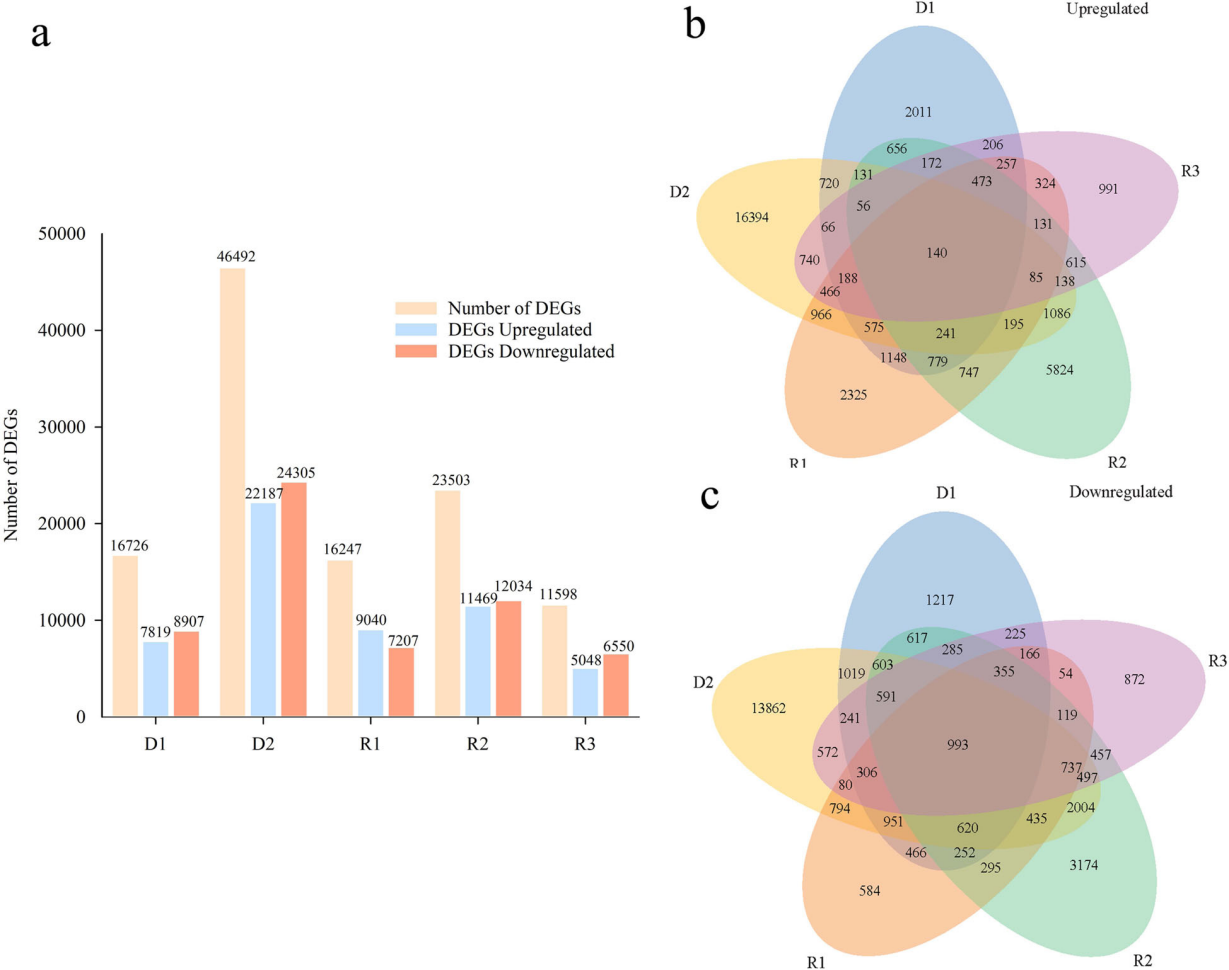
Up- and downregulated DEGs and venn diagrams showing the numbers of DEGs across five comparisons. **a**: DEGs were upregulated or downregulated by drought-rehydration treatment in Giant Juncao. D1, D2, R1, R2, and R3 show the comparisons of differential unigenes identified between 7 days of drought vs. control, 14 days of drought vs. control, 1 day of rehydration vs. control, 5 days of rehydration vs. control, and 9 days of rehydration vs. control, respectively; **b**: upregulated DEGs in venn diagrams; **c**: downregulated DEGs in venn diagrams

The gene expression levels were calculated and normalized using the TMM method; |log2(FoldChange)| > 1 and adjusted *p*-value < 0.05 were set as the threshold for significant differential expression. We found 62,298 unigenes which showed differential expression in drought-rehydration-treated samples. In total, 16,726 and 46,492 DEGs were detected at days 7 and 14 of drought treatment. A total of 7819 and 22,187 DEGs were upregulated, whereas 8907 and 24,305 DEGs were downregulated at D1 and D2, respectively (Fig. 3a). Compared with control plants, a total of 16,247 (9040 upregulated and 7207 downregulated), 23,503 (11,469 upregulated and 12,034 downregulated), and 11,598 (5048 upregulated and 6550 downregulated) DEGs were detected at R1, R2, and R3, respectively (Fig. 3a).

To identify common unigenes in different physiological stages, the overlaps in each comparison were shown in a Venn diagram (Fig. 3b and c). The highest overlap of the up- and downregulated genes was between 14 days of drought and control, which showed > 70% common unigenes, suggesting plant damage after strong drought (Figs. 3b and c). The second highest overlap of the up- and downregulated unigenes was between 5 days of rehydration, suggesting a gradual recovery after re-watering at R2 (Figs. 3b and c). A total of 140 upregulated unigenes (Fig. 3b) and 993 downregulated unigenes (Fig. 3c) overlapped with those of 7 and 14 days of drought and 1, 5, and 9 days of rehydration versus control, respectively, suggesting that a shared group of genes was associated with response to water deficit and rehydration treatment conditions.

### GO enrichment in DEGs

To identify the major biological processes that are expressed at the drought-rehydration conditions, we showed each GO enrichment analysis of up- and downregulated DEGs at five physiological stages depending on FDR < 0.05 (Additional file 2). On the basis of the GO enrichment results, we selected the most significant accession to explain the physiological changes. Processes that promote activity were mainly enriched in the downregulated DEGs at D1, whereas only the carbohydrate metabolic process was enriched in the downregulated DEGs at D2 and after re-watering at R1. Drug binding and ATP binding processes were enriched in the downregulated DEGs at D2 and were further enriched at R2, suggesting reduced protein combining ability in response to drought. Ribosome and peptide metabolic processes were enriched in the upregulated DEGs at R1 and R2, whereas the structural constituent of the ribosome was enriched in the upregulated and downregulated DEGs at R3, suggesting the gradual completion of cell repair. DNA-binding TF activity and transcription regulator activity were enriched in the upregulated DEGs, whereas the cellular protein metabolic process was enriched in the downregulated DEGs at R3, suggesting improvement in TF activity and decreased protein degradation after re-watering (Table 2).

**Table 2:** GO analysis of the most significant up- and downregulated DEGs (FDR < 0.05)

	Up-regulated				Down-regulated			
Comparison	GO accession	Up-Description	Corrected *p* value	DEGs number	GO accession	Down-Description	Corrected *p* value	DEGs number
D1	GO:0140098	catalytic activity, acting on RNA	2.76E-05	137	GO:0004672	protein kinase activity	4.48E-38	492
	GO:0008168	methyltransferase activity	0.0098431	56	GO:0006468	protein phosphorylation	6.47E-38	484
	GO:0016741	transferase activity, transferring one-carbon groups	0.0098431	61	GO:0016773	phosphotransferase activity, alcohol group as acceptor	2.61E-33	511
	GO:0006801	superoxide metabolic process	0.011936	10	GO:0016301	kinase activity	6.61E-31	521
	GO:0004719	protein-L-isoaspartate (D-aspartate) O-methyltransferase activity	0.011936	16	GO:0016310	phosphorylation	1.33E-30	501
D2	GO:0005975	carbohydrate metabolic process	0.031471	366	GO:0008219	cell death	1.29E-13	352
					GO:0008144	drug binding	1.29E-13	1615
					GO:0006915	apoptotic process	2.19E-13	336
					GO:0012501	programmed cell death	2.19E-13	336
					GO:0005524	ATP binding	2.21E-13	1593
R1	GO:0003735	structural constituent of ribosome	1.80E-14	229	GO:0005975	carbohydrate metabolic process	6.27E-08	209
	GO:0005840	ribosome	1.80E-14	229	GO:0004194	obsolete pepsin A activity	1.03E-07	37
	GO:1990904	ribonucleoprotein complex	6.26E-11	242	GO:0044262	cellular carbohydrate metabolic process	8.70E-07	71
	GO:0005198	structural molecule activity	4.53E-09	273	GO:0016798	hydrolase activity, acting on glycosyl bonds	8.57E-06	106
	GO:0006518	peptide metabolic process	1.34E-07	279	GO:0004553	hydrolase activity, hydrolyzing O-glycosyl compounds	2.33E-05	102
R2	GO:0003735	structural constituent of ribosome	9.55E-28	316	GO:0008144	drug binding	4.30E-42	1102
	GO:0005840	ribosome	9.55E-28	316	GO:0005524	ATP binding	4.30E-42	1092
	GO:0006412	translation	1.79E-24	402	GO:0030554	adenyl nucleotide binding	4.30E-42	1094
	GO:0006518	peptide metabolic process	3.43E-24	404	GO:0032559	adenyl ribonucleotide binding	4.30E-42	1094
	GO:0043043	peptide biosynthetic process	3.43E-24	404	GO:0006468	protein phosphorylation	9.13E-41	618
R3	GO:0003700	DNA-binding transcription factor activity	2.68E-05	109	GO:0003735	structural constituent of ribosome	2.54E-18	216
	GO:0140110	transcription regulator activity	0.00017175	109	GO:0005840	ribosome	2.54E-18	216
	GO:0016759	cellulose synthase activity	0.00061948	16	GO:0044267	cellular protein metabolic process	2.38E-17	583
	GO:0016760	cellulose synthase (UDP-forming) activity	0.00061948	16	GO:1990904	ribonucleoprotein complex	8.79E-16	230
	GO:0030243	cellulose metabolic process	0.00061948	16	GO:0043228	non-membrane-bounded organelle	1.38E-13	260

### KEGG pathway enrichment analysis

**Fig. 4:**
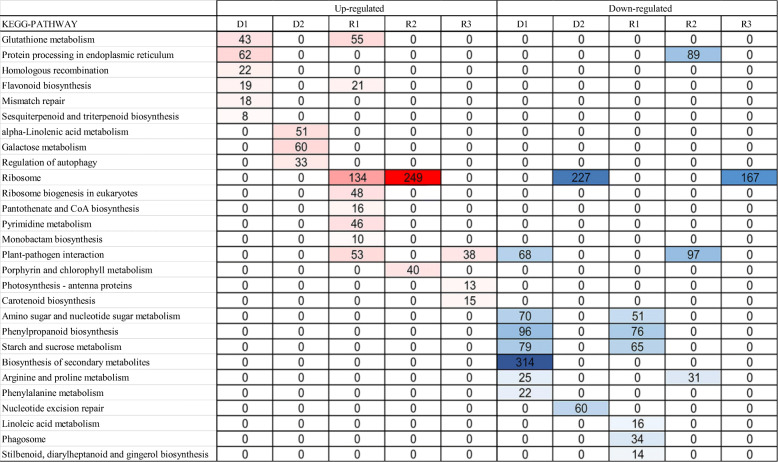
Heatmap diagram reflecting the dynamics of enriched biological processes in KEGG analysis. Drought-rehydration-related physiological categories over the course of the experiment as obtained from KEGG enrichment (see Additional file 3). Enriched processes with FDR < 0.05 were included in the heatmap. Red and blue colors represent upregulated and downregulated DEGs, respectively. The intensity of the color reflects the number of DEGs as indicated at each physiological stage

The KEGG pathway enrichment analysis showed that 1972 upregulated and 2337 downregulated DEGs were involved in 117 different pathways in Giant Juncao when comparing 7 days of drought with the control (Tables S3 and Additional file 3). With prolonged drought, 4490 upregulated and 5725 downregulated DEGs were annotated in 119 and 116 different pathways (Tables S3 and Additional file 3). After rehydration for 1, 5, and 9 days, 2166, 3106, and 1126 upregulated DEGs and 2096, 3229, and 2016 downregulated DEGs were annotated in the KEGG pathway database, which involved 111, 122, and 107 different upregulated pathways and 115, 115, and 114 different downregulated pathways (Tables S3 and Additional file 3). According to the KEGG pathway analyses, a heatmap reflecting the different biological processes in the significantly enriched pathways was constructed (Fig. 4). In these pathways, glutathione metabolism was enriched at D1 and R1 in the upregulated DEGs, whereas protein processing in the endoplasmic reticulum was enriched at D1 and in the downregulated DEGs at R2. Pathways related to homologous recombination and mismatch repair were enriched at D1, whereas alpha-linolenic acid metabolism, galactose metabolism, and regulation of autophagy were enriched at D2 in upregulated DEGs, suggesting improved metabolism in response to drought. Ribosome biogenesis in eukaryotes, pantothenate and CoA biosynthesis, and pyrimidine metabolism were enriched in the upregulated DEGs at R1, reflecting recovery after re-watering. The major of pathway related to ribosome was enriched in the upregulated DEGs at R1 and R2 and downregulated DEGs at D2 and R3. Plant pathogen interaction was enriched in the upregulated DEGs at R1 and R3 and downregulated DEGs at D1 and R2. The upregulated DEGs related to photosynthesis were enriched in porphyrin and chlorophyll metabolism, photosynthesis - antenna proteins, and carotenoid biosynthesis pathways at R2 and R3, suggesting that photosynthesis plays an important role after re-watering. Amino sugar and nucleotide sugar metabolism, phenylpropanoid biosynthesis, and starch and sugar metabolism were enriched in the downregulated DEGs at D1 and R1, whereas arginine and PRO metabolism was enriched at D1 and R2. Biosynthesis of secondary metabolism, phenylalanine metabolism, and nucleotide excision repair were enriched in the downregulated DEGs at D1 and D2. The pathways related to linoleic acid metabolism; phagosome; and stilbenoid, diarylheptanoid, and gingerol biosynthesis were enriched in the downregulated DEGs at R1 (Fig. 4).

### Response of key unigenes to photosynthesis, antioxidant, osmoregulation and plant hormone signal transduction processes

To further understand the response of photosynthesis, antioxidant, osmoregulation and plant hormone signal transduction processes to drought and recovery processes, annotated DEGs with fpkm > 2 and verified functions related to six pathways (including carbon fixation in photosynthetic organisms, photosynthesis, photosynthesis - antenna proteins, peroxisome, arginine and PRO metabolism and plant hormone signal transduction) were selected and grouped into clusters (Table 3, 4 and 5). In Giant Juncao under D1, 4, 7, and 2 genes related to the carbon fixation in photosynthetic organisms, photosynthesis, and photosynthesis - antenna proteins were upregulated, whereas 23, 4, and 2 genes were downregulated, respectively (Additional file 4). These genes mainly included encoding alanine aminotransferase 2 (ALT2) (c116433_g3_i1), alanine aminotransferase (ALT) (c111450_g1_i1), phosphoenolpyruvate carboxylase (PPC) (c114318_g2_i1, c119399_g3_i1, c98555_g1_i1), and photosystem II protein M (PsbM) (c47941_g1_i1), which were downregulated, and photosystem I P700 apoprotein A1 (PsaA) (c8283_g1_i1), which was upregulated. With the increasing degree of drought stress, the genes encoding aspartate aminotransferase (AST) (c116017_g2_i1), fructose-bisphosphate aldolase cytoplasmic isozyme (FBA) (c112618_g1_i3), malate dehydrogenase (MDH) (c101919_g2_i1, c112985_g2_i3, c112985_g2_i2), triosephosphate isomerase (TPI1) (c111215_g2_i3), ribose-5-phosphate isomerase (RPI) (c118707_g1_i7), and aldolase C-2 (ALDOC-2) (c75858_g1_i1) were upregulated, and the |log2(FoldChange)| was between 1.5 and 4.1 at D2. However, the |log2(FoldChange)| of the downregulated genes was between 1.1 and 12.5, especially the genes (c111709_g1_i1, c112618_g1_i1, c117517_g1_i1, c116040_g1_i1) whose |log2(FoldChange)| is more than 9; these genes separately encoded chloroplast fructose-1,6-biphosphate aldolase (CpFBA), fructose-bisphosphate aldolase (FDA), P-pyruvate carboxylase (PEPC), and chloroplast ribulose-1,5-bisphosphate carboxylase/oxygenase small subunit (rbcS-Ma5) (Table 3).

**Table 3:**
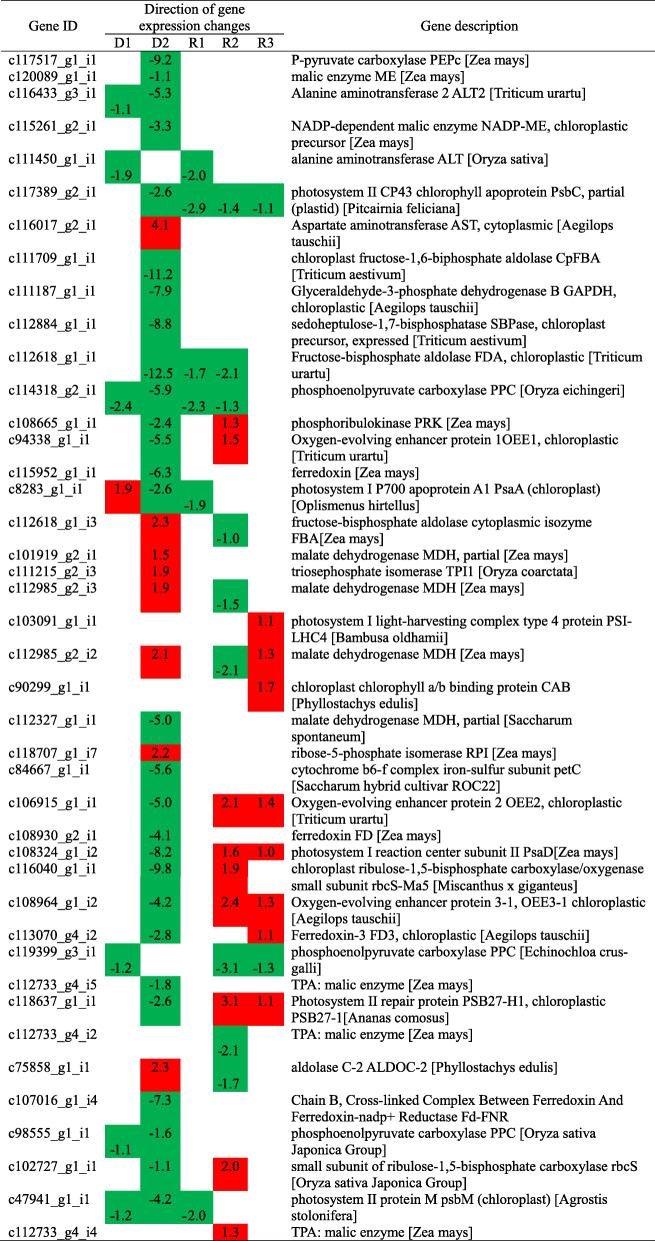
Selected DEGs involved in the photosynthesis during drought and rehydration of Giant Juncao.

The number shows the log_2_(FoldChange). Red and green colors indicate up- and downregulated DEGs, respectively.

**Table 4:**
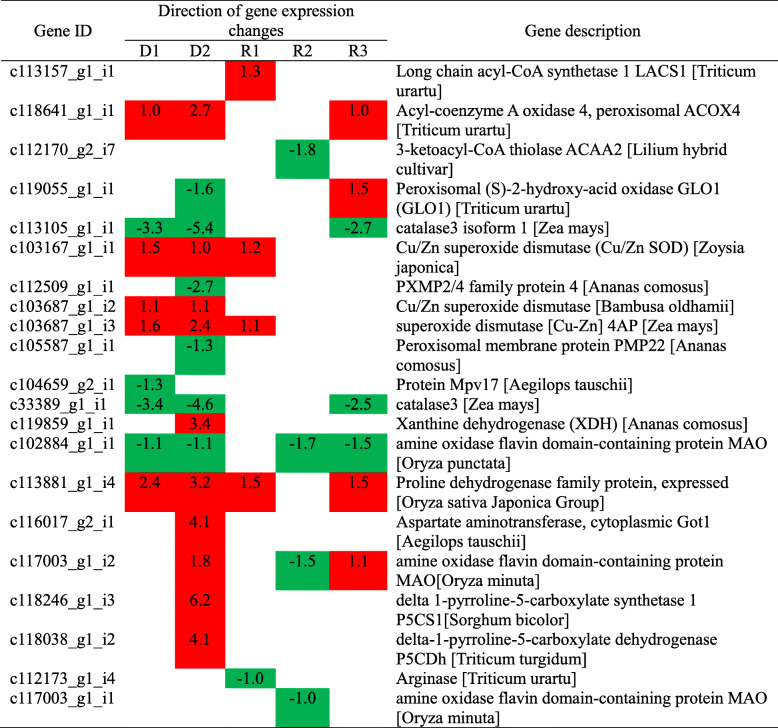
Selected DEGs involved in peroxisome and arginine and proline metabolism of Giant Juncao.

The number shows the log_2_(FoldChange). Red and green colors indicate up- and downregulated DEGs, respectively.

**Table 5:**
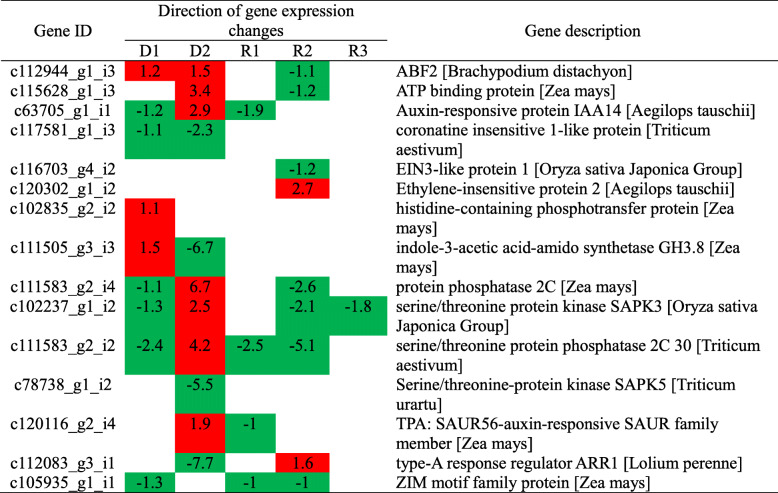
Selected DEGs involved in hormone signal transduction during drought stress and rehydration.

The number shows the log_2_(FoldChange). Red and green colors indicate up- and downregulated DEGs, respectively.

After rehydration, all genes were downregulated, and the |log2(FoldChange)| was between 1.7 and 2.9 at R1. With the extension of rehydration time, more genes became upregulated at R2, especially those encoding phosphoribulokinase (PRK) (c108665_g1_i1), oxygen-evolving enhancer protein 1 (OEE1) (c94338_g1_i1), oxygen-evolving enhancer protein 2 (OEE2), photosystem I reaction center subunit II (PsaD) (c108324_g1_i2), rbcS-Ma5 (c116040_g1_i1), oxygen-evolving enhancer protein 3–1 (OEE3–1), photosystem II repair protein (PSB27-H1), small subunit of ribulose-1,5-bisphosphate carboxylase (rbcS) (c102727_g1_i1), and TPA:malic enzyme (c112733_g4_i4) (Table 3). At the end of the rehydration, photosystem II CP43 chlorophyll apoprotein (PsbC) and PPC, which were encoded by c117389_g2_i1 and c119399_g3_i1, were downregulated, whereas other genes were upregulated (Table 3).

Antioxidant and osmoregulation processes play an important role in coping with drought stress. With the aggravation of drought stress, peroxisomal (S)-2-hydroxy-acid oxidase GLO1 (GLO1) (c119055_g1_i1) was downregulated at D2 and upregulated at R3. Cu/Zn SOD (c103167_g1_i1) and SOD [Cu-Zn] 4AP (c103687_g1_i3) were upregulated at D1, D2, and R1 but disappeared at R2 and R3 (Table 4). c104659_g2_i1, c112509_g1_i1, and c105587_g1_i1, which encoded protein Mpv17, PXMP2/4 family protein 4, and peroxisomal membrane protein PMP22, respectively, were downregulated at D1 and D2, whereas xanthine dehydrogenase (XDH) (c119859_g1_i1) was only upregulated at D2. After rehydration, long-chain acyl-CoA synthetase 1 (LACS1) (c113157_g1_i1) was only upregulated at rehydration stages, whereas 3-ketoacyl-CoA thiolase (ACAA2) was downregulated at R2 (Table 4). For the arginine and PRO metabolism pathway, AST, cytoplasmic (Got1) (c116017_g2_i1), delta 1-pyrroline-5-carboxylate synthetase 1 (P5CS1) (c118246_g1_i3), and delta-1-pyrroline-5-carboxylate dehydrogenase (P5CDh) (c118038_g1_i2) were upregulated at D2, whereas arginase (c112173_g1_i4) and amine oxidase flavin domain-containing protein (MAO) (c117003_g1_i2, c117003_g1_i1) were downregulated at R1 and R2, respectively (Table 4).

Plant hormones are secondary metabolites in plants. In response to drought stress, a large number of upregulated and downregulated DEGs are expressed in plant hormone signal transduction, especially at 14 days of drought stress (Additional file 5). With the intensification of drought stress, coronatine-insensitive 1-like protein (c117581_g1_i3), serine/threonine-protein kinase SAPK5 (c78738_g1_i2) and type-A response regulator ARR1 (c112083_g3_i1) were downregulated, while the ABF2 (c112944_g1_i3), ATP binding protein (c115628_g1_i3), histidine-containing phosphotransfer protein (c102835_g2_i2), TPA: SAUR56-auxin-responsive SAUR family member (c120116_g2_i4) were upregulated at D2. After rehydration, ethylene-insensitive protein 2 (c120302_g1_i2) and type-A response regulator ARR1 (c112083_g3_i1) were upregulated at R2, while ABF2, ATP binding protein, and TPA: SAUR56-auxin-responsive SAUR family member exhibited the opposite of what is expressed during drought conditions (Table 5).

### Classification of TFs in Giant Juncao under drought stress and rehydration treatment

**Fig. 5:**
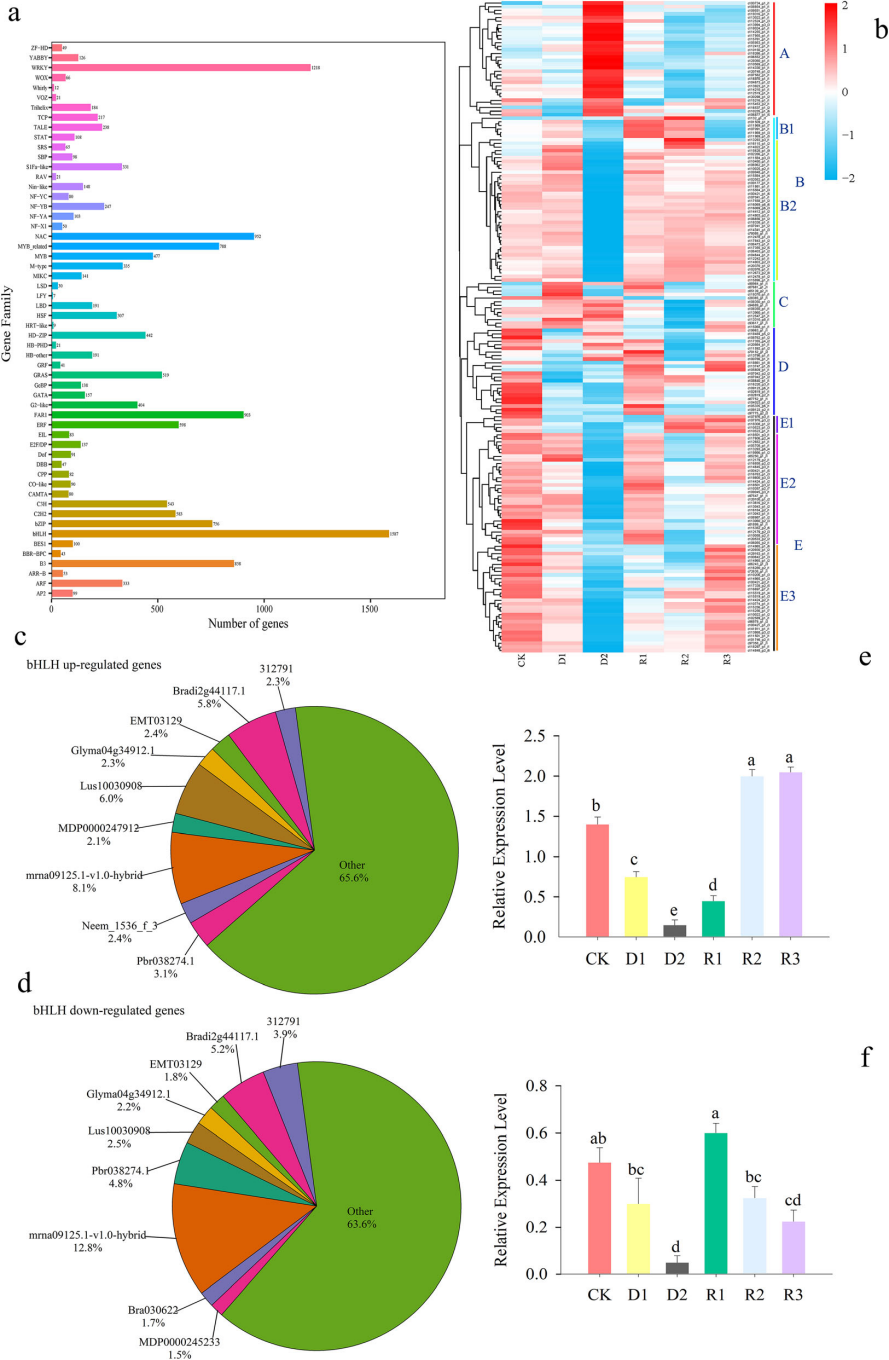
TFs that were differentially expressed under drought and rehydration conditions in Giant Juncao. **a**: major TF families; **b**: hierarchical clustering heatmap of 181 bHLH which located at high level of expression (FPKM ≥20); **c**: the most dominant bHLH with upregulated TFs; **d**: the most dominant bHLH with downregulated TFs; **e**: qRT-PCR analysis the relative expression level of c104644_g1_i1; **f**: qRT-PCR analysis the relative expression level of c109045_g3_i1

The TFs of 56 families were included in the DEGs during drought and rehydration (Additional file 6). The largest group of TFs was the basic helix-loop-helix (bHLH) family, followed by the WRKY, whereas other TFs belonged to the NAC, FAR1, B3, MYB-related, and basic leucine zipper (bZIP) families (Fig. 5a). During drought and rehydration treatments, 181, 432, 295, 251, and 114 TFs among the 510, 1090, 521, 642, and 301 TFs in the bHLH family were upregulated at D1, D2, R1, R2, and R3, respectively (Fig. 5a). The 91 upregulated and 281 downregulated TFs in the WRKY family increased to 398 upregulated and 497 downregulated TFs during drought stress. After rehydration, the numbers of upregulated TFs in the WRKY family tended to decrease, whereas those of downregulated TFs tended to increase and then decrease (Additional file 6).

At a high expression level (FPKM ≥20), 181 bHLH were used for hierarchical clustering analysis in water deficit and rehydration conditions (Fig. 5b). These bHLH were grouped into five cluster from A to E. The 32 bHLH in cluster A were highly expressed at D2 but have low expression in other stages. In cluster B, 6 and 40 bHLH were in B1 and B2, respectively. The gene c1132_g1_i1located in B1 were highly expressed at R1 and R2. Some genes with low expression were found at D2 in cluster B2. Cluster C consists of 13 members that were highly expressed from CK to D2 but had low expression at R1 to R3. In cluster D, 24 genes were expressed from high to low then back to high expression during drought and rehydration conditions. Cluster E was divided into three subclusters, namely, E1, E2, and E3, which had 5, 31, and 30 genes, respectively. In E1, c107976_g3_i2 and c110523_g1_i1 had the highest expression level at CK, R2 and R3 (Fig. 5b). However, the most dominant bHLH, with 68 upregulated and 118 downregulated TFs, was mrna09125.1-v1.0-hybrid (Figs. 5c and d).

To validate the accuracy of TF sequencing, the relative expression level of two randomly selected bHLH was examined using quantitative RT-PCR analysis (Fig. 5e and f). After water deficit, the relative expression level of c104644_g1_i1 and c109045_g3_i1 decreased significantly over 14 days of drought and then increased during 1 day of recovery. These results demonstrated similar expression patterns to the results of RNA-Seq.

### Weighted gene co-expression network analysis module generation and functional enrichment analysis

**Fig. 6:**
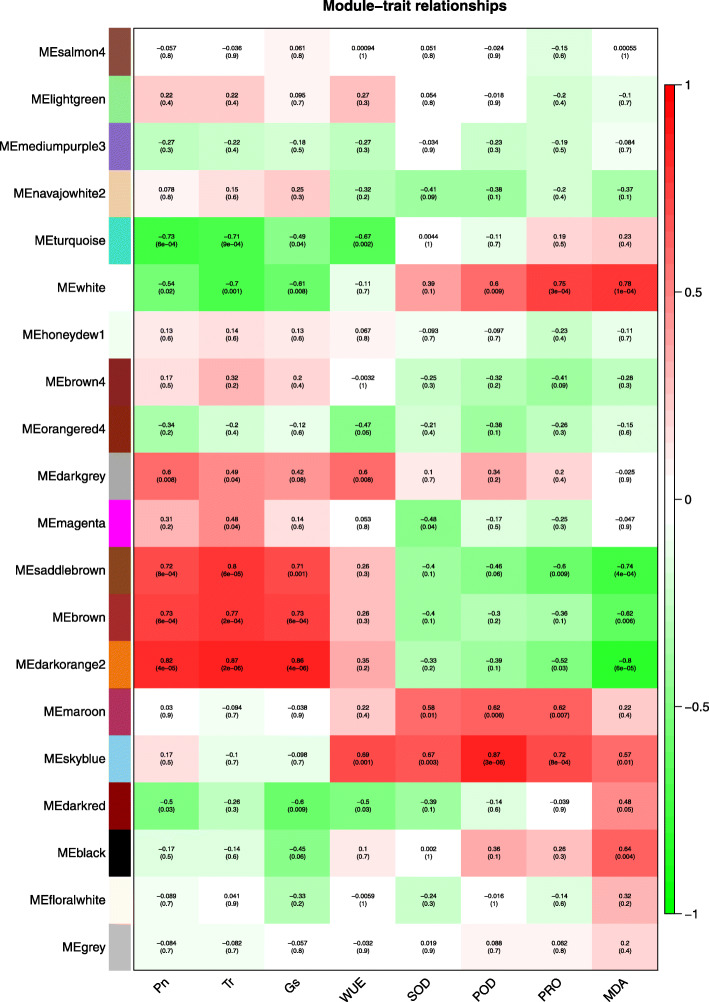
Module-trait relationship with physiological indexes. The number represents the correlation coefficient about modules with physiological indexes. The number in the bracket means *p-value*

The gene co-expression profiles of Giant Juncao in response to water deficit and rehydration conditions were analyzed by using WGCNA to detect the relationship between genes and physiological indexes, as well as inter- or intramodular genes. Twenty co-expression modules and correlation coefficients were identified and obtained (Fig. 6). The physiological indexes were positively correlated with the darkorange2, skyblue, and white modules, and the correlation coefficient was between 0.67–0.87. However, Pn and Tr were negatively correlated with turquoise module, MDA was negatively correlated with darkorange2 module, and the correlation coefficient was 0.71–0.8.

**Fig. 7:**
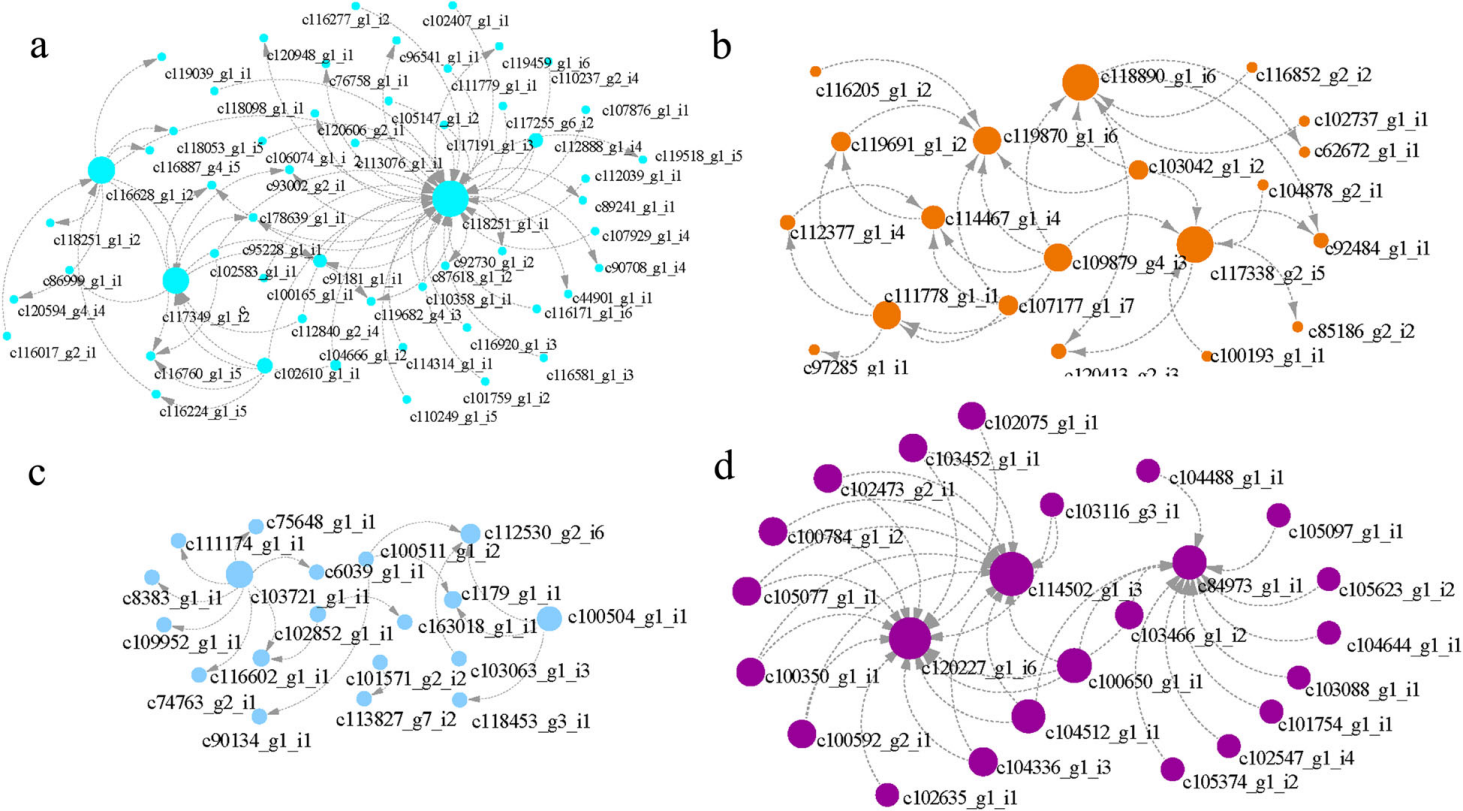
Net-work analysis the hub genes in four modules which had high correlation coefficient (0.67–0.87) with physiological indexes. **a**: turquoise module; **b**: darkorange2 module; **c**: skyblue module; **d**: white module. Node size means the degree of genes

The genes related to the physiological indexes of Giant Juncao were mainly concentrated in the turquoise, darkorange2, skyblue, and white modules. Therefore, the genes with a higher weight in each module were selected for analysis and for drawing the network. The genes, including c118251_g1_i1, c118890_g1_i6, c103721_g1_i1, and c120227_g1_i6, which encoded β-glucosidase, peptide/histidine transporter, LOC103649875, and α/β hydrolase, respectively, had the highest degree in four modules (Fig. 7, Additional file 7).

## Discussion

Drought tolerance is a complex biological process that is controlled and adjusted by several genes in the plant. In this study, a comprehensive method was used to explore the molecular mechanism of physiological response during drought stress and recovery in Giant Juncao, a species without a reference genome. High-throughput sequencing (RNA-seq) and de novo transcriptome analysis [27] were used to investigate the molecular response of Giant Juncao to drought stress.

### Insights into the de novo transcriptome assembly and sequence annotation

Due to the lack of genomic and transcriptome data, the genetic background and functional genes of Giant Juncao are not completely clear. Therefore, a large number of unigenes have not been annotated, and only 61.7% of the total unigenes were annotated in at least one database. This is different to the annotation results of transcriptome sequencing of reported species, such as common vetch, which has approximately 500 million clean reads employed to assemble 174,636 transcripts and 83.48% unigenes annotated in at least one database [28]. In the common buckwheat, 53,404 unigenes were assembled, and 39.33% unigenes were annotated in the GO database [29], whereas 8426 and 12,642 unigenes of *Taxus* species were annotated in the KEGG and GO database, respectively [30]. This indicates that for those species without a reference genome, a large amount of data in the transcriptome still needs to be further mined.

### Response of photosynthesis and related processes to drought and rehydration conditions

Through the analysis of transcriptome data, some genes in the photosynthetic pathway showed difference in abundance, which may cause the photosynthetic indices in Giant Juncao to change after no irrigation and rehydration (Fig. 1b). At the beginning of drought stress (D1), the WUE showed a significant upward trend (Fig. 1b). WUE is a comprehensive reflection of photosynthetic and transpiration characteristics of plants. During water deficit, the decrease rate of Pn was slower than that of Tr, so the WUE exhibited an upward trend. However, according to the analysis of transcriptome data, only PsaA showed an upregulated expression (Table 3). PsaA is a core protein and crucial for the functional assembly of PSI [31]. The upregulation of PsaA will keep PSI stable to delay the decline of the photosynthetic rate. Thus, the gene encoding PsaA could adjust higher WUE to cope with light drought stress. Pn decreased significantly with prolongation of stress, which may be caused by the downregulation of PEPC, CpFBA, FDA, and rbcS-Ma5 proteins to cope with drought stress (Fig. 1b and Table 3). PEPC is an important C_4_ plant photosynthesis multifunctional enzyme, which can be used to transfer into plants to improve photosynthetic efficiency, and becomes an important way to improve crop yield [32–35]. Under drought stress, PEPC transgenic rice showed strong drought and light tolerance ability because it can accumulate malic acid or oxaloacetic acid in its guard cells to promote the opening of stomata and enhance photosynthesis [32]. In our study, when the Giant Juncao experienced strong water deficit, the PEPC protein was downregulated, causing Pn, Tr, and Gs to decline. These results showed that downregulated PEPC will cause stomatal closure, which decreases Tr and leads to the decline in Pn with less CO_2_ into the mesophyll cell. CpFBA is one of the key enzymes controlling the rate of photosynthesis by improving carbon fixation efficiency in the Calvin cycle to enhance the resistance of plants [36, 37]. It plays very important roles in improving the adaptability of *Sesuvium portulacastrum* under salt and drought stress [38] and maintaining the high photosynthetic rate and biomass of transgenic tobacco [39, 40]. In the present study, drought caused the downregulated expression of several key enzyme regulatory genes in photosynthesis, which was also a major factor in the significant decline in Pn of Giant Juncao.

However, at the beginning of re-watering, Pn, Tr, and WUE significantly increased, whereas Gs remained stable. Meanwhile, the expression of genes was downregulated (Fig. 1b and Table 3). This finding reflects that plants cannot quickly recover from drought stress because the stoma does not open immediately. During rehydration for 5 to 9 days, an increasing number of genes that encoded PRK, OEE1, PSI-LHC4, MDH, CAB, OEE2, psaD, rbcS-Ma5, OEE3–1, FD3, PSB27–1, rbcS, and TPA:malic enzymes were upregulated (Table 3). These protein-encoding genes could be the major regulatory genes related to recovery from drought stress. When Giant Juncao was rehydrated again, WUE could reach the control state (Fig. 1b). According to the transcriptome results, the OEE protein family-encoding genes were upregulated. OEEs are the most important proteins for oxygen evolution and photosystem II stability [41]. In our study, the OEE genes played an important role in the process of drought and rehydration of Giant Juncao. The results were similar to those of cultivar wheat and Norway spruce, wherein OEEs were the key enzymes in the photosynthesis system and enhanced in response to drought stress [42, 43].

### Antioxidant enzyme and osmotic adjustment response to drought and re-watering conditions of Giant Juncao

Giant Juncao improved its adaptability to cope with drought environment by regulating the ability of antioxidants and the quantity of osmoregulation substances. In this study, SOD, POD, and PRO initially increased under drought stress and then decreased (Fig. 1b). After rehydration, POD and PRO continued to decrease, whereas SOD showed the opposite trend. SOD is a kind of metal enzyme that widely exists in plants. According to the different metal subgroups, SOD is usually divided into three different types, for example, Cu/Zn-SOD, Mn-SOD, and Fe-SOD [44]. In our study, Cu/Zn SOD was the major regulation protein in coping with drought stress. Cu/Zn-SOD is the first-line antioxidant system to remove reactive oxygen and is closely related to plant stress resistance [45]. Peroxisomes are vital cell organelles and may contain highly variable sets of enzymes that adjust many important cellular processes [46]. MPV17 is one of the import proteins in the peroxisomal membrane in eukaryotes [47]. In our study, MPV17 was downregulated under drought stress and disappeared during recovery (Table 4). This finding is probably because this gene acts as a major regulator in the formation of antioxidant enzymes during water shortage conditions. During drought stress, PRO was probably upregulated through P5CS1, which supports the function of PRO as an osmoregulator [48].

### Plant hormone reflect drought and rehydration condition of Giant Juncao

Plant hormones not only widely participate in the various growth and development stages of plants but also play an important role in regulating plant growth to adapt to various kinds of biological or abiotic stress. In our research, ABF2, ATP binding protein, TPA: SAUR 56-auxin-responsive SAUR family member, and type-A response regulator ARR1 l showed opposite expression under drought and recovery processes (Table 5). ABF2, as an ABRE-binding factor, is a principal regulator of the ABA-dependent pathway [49]. When Giant Juncao was subjected to drought stress, ABF2 was upregulated at D1 and D2. However, when the dehydration state disappeared, the ABF2 became downregulated at R2, thereby showing that ABF2, as a hormone regulator, can rapidly regulate plant growth to adapt to environmental stress changes (Table 5). A similar result has been reported with Arabidopsis and rice, in which the overexpression of ABF2 significantly increased the drought tolerance of plants [14, 50]. This finding also confirms that ABF2 plays an important role in plant response to drought stress.

### TFs regulate the drought resistance of Giant Juncao

Transcription regulation is a key step in plant response to stress by temporarily and spatially regulating their target genes of transcription [51, 52]. TFs plays an important role in plant drought resistance via the transcriptional regulation of downstream genes to improve plant stress resistance [53]. Thus, this study provides important information for discovering and separating TFs and further elucidating the molecular mechanisms underlying drought tolerance in Giant Juncao. More than 15,000 DEGs encoding TFs were distributed in the major TF families, including bHLH, WRKY, NAC, MYB-related, FAR1, B3, and bZIP families (Fig. 5a and Additional file 6). These identified TF families are well known in stress tolerance in plants [28].

The bHLH family is one of the largest TFs in plants and play critical roles in light signaling, hormone signaling, wound, and drought stress response [54]. For example, the bHLH family has 183, 231 and 571 members in the rice, maize and wheat genomes respectively [55], 162 members in *Arabidopsis* [56], 230 members in Chinese cabbage [57], 146 members in *Brachypodium distachyon* [58], and 175 members in apple [59] that have been identified, and most of them have been characterized with drought stress responses [60]. In addition, a rice domain gene (*OsbHLH148*) can provide drought tolerance as a jasmonate signaling module component [61]. However, the overexpression of wheat *TabHLH39* genes enhances drought resistance, salt tolerance, and frost resistance in transgenic *Arabidopsis* [62]. In our study, drought stress induced the upregulation of 843 DEGs and downregulation of 923 DEGs belonging to the bHLH TF gene family (Additional file 6). Moreover, 432 and 658 DEGs were upregulated and downregulated, respectively, after 14 days of drought (Additional file 6). After rehydration, the number of bHLH family members declined (Additional file 6). This finding indicates that the genes regulated by bHLH TF will change significantly under aggravated drought conditions, which proves that bHLH TF plays an active role in the regulation of drought stress.

For the cluster analysis, we divided bHLH into 5 categories and 8 subcategories, and the genes in each subcluster showed similar expression trends (Fig. 5b). Thirty-two bHLH located in cluster A were upregulated under severe drought condition (D2) but were downregulated after rehydration (Fig. 5b). The expression of genes in cluster B was reversed in cluster A, thereby indicating that bHLH located in clusters A and B had opposite functions during the corresponding drought stress and rehydration conditions of Giant Juncao. This result might be due to the large number of bHLH genes and their powerful functions, thereby causing them to have different expressions.

### Network analysis revealed the key genes of drought resistance of Giant Juncao

WGCNA analysis revealed the hub genes in each module after the four key modules related to drought resistance of Giant Juncao were identified (Figs. 6, 7 and Additional file 7). In the turquoise module, β-glucosidase was the hub gene related to hormone regulation. Moreover, it was upregulated and downregulated when Giant Juncao experienced severe drought stress and was repaired after rewatering (Additional file 7). β-glucosidase recycling, as a key step in determining ABA concentration, affects drought tolerance and photosynthesis. β-glucosidase isoenzyme contributes significantly to cellular ABA pools and plays an important part in stomatal density and aperture size [63]. In *Arabidopsis thaliana* leaves, inactive ABA was hydrolyzed by β-glucosidase, and then a large amount of ABA accumulated in the leaves to improve its drought resistance [64]. The result was similar to those of this paper because β-glucosidase was highly expressed under drought stress.

## Conclusions

In this study, we report the first transcriptome data for the characterization of drought-rehydration-related genes in Giant Juncao. A total of 93,907 unigenes were de novo assembled, and 57,941 genes were annotated by functional databases. The expression of DEGs and the identification of the hub gene encoding β-glucosidase can help elucidate the response mechanism, rehydration recovery, and physiological indices of plants during drought stress. This study provides not only insights into the genomics of adapting drought tolerance in Giant Juncao but also candidate genetic resources for abiotic stress resistance of plant development.

## Methods

### Plant material and drought-rehydration treatment

Giant Juncao seedlings were collected from the plantation base in Fujian Agriculture and Forestry University. Plants with full sprouts and consistent stem thickness were selected and planted in pots filled with 1:1 turfy soil and vermiculite. Each pot contained 2.5 kg of turfy soil and vermiculite with one stem node of Giant Juncao. All materials were planted in a greenhouse at room temperature (27 °C) and well irrigated. One month later, plants with consistent (seven leaves) and healthy growth were selected for drought experiment.

Drought stress was imposed on the plants by stopping irrigation. Seedlings were sampled by collecting their third and fourth leaves at day 0 for control and at days 7 and 14 (D1 and D2) during drought. After 14 days of drought stress, the seedlings were watered to field capacity, and leaf tissue was collected after 1, 5, and 9 days (R1, R2, and R3) following re-watering. To eliminate the influence of the development process on the test results, a unified end time of the experiment was set, and the sampling was unified. Four independent biological replicates were collected for ecological and physiological tests and three replicates were used for RNA-Seq analysis. The tissues were collected immediately into liquid nitrogen and stored at − 80 °C until used.

### Measurement of the ecological and physiological indices of Giant Juncao

In this study, a CIRAS-3 portable photosynthetic apparatus (PP-Systems Company, Amesbury, MA01913, USA) was used to observe the photosynthetic parameters of the seedlings. The CO_2_ concentration in the reference leaf chamber was controlled at a constant value of 390 μmol·mol^− 1^, and the photosynthetically active radiation and air relative humidity were set to 1200 μmol m^− 2^ s^− 1^ and 75%, respectively. The experimental apparatus automatically recorded the net photosynthetic rate (Pn, μmol m^− 2^ s^− 1^), transpiration rate (Tr, mol m^− 2^ s^− 1^), and stomatal conductance (Gs, μmol m^− 2^ s^− 1^). Meanwhile, a formula was used to calculated the water use efficiency (WUE) as follows: WUE = Pn/Tr, mmol mol^− 1^. The physiological indices, including superoxide dismutase (SOD), peroxidase (POD) activity, and malondialdehyde (MDA) and proline (PRO) contents, were determined in accordance with a previously reported method [65, 66].

### RNA extraction

High quality total RNA was isolated and extracted from the Giant Juncao seedling using the TRIzol reagent (TransGen, Beijing, China) according to the product instructions. RNA purity and concentration were tested using a NanoDrop 1000 spectrophotometer (Thermo Fisher Scientific, Wilmington, DE, USA). The integrity of RNA molecules was measured using an Agilent Bioanalyzer 2100 system (Agilent Technologies, Santa Clara, CA, USA). Total RNA quality was detected using 1% agarose gels.

### Sequencing library preparation for transcriptome

The RNA-seq library was constructed with Illumina’s TruSeq RNA Sample Preparation Kit (Illumina Inc., San Diego, CA, USA) according to the product instruction. mRNA connected with poly-T oligo-attached magnetic beads was purified from total RNA, and divalent cations were used to conduct fragmentation under high temperatures in NEBNext First Strand Synthesis Reaction Buffer (5×). Random hexamer primers and M-MuLV Reverse Transcriptase (RNase H-) were used for first-strand cDNAs from the reverse transcribed fragmented mRNA. Then, the DNA Polymerase I and RNase H (Invitrogen, Carlsbad, CA, USA) were used to synthesize the second-strand cDNAs. The remaining moleculars were converted into blunt ends by exonuclease/polymerase activities. After purification with the AMPure XP system (Beckman Coulter, Pasadena, CA, USA), the cDNA fragments were resolved in elution buffer for end reparation and addition of a poly(A) tail and then connected to sequencing adaptors with suitable length fragments. Libraries were sequenced on an Illumina HiSeq 4000 platform to generate paired-end reads of 150 bp.

### Sequence read mapping and assembly

Initial processing of original data in FASTQ file was achieved. Bowtie 2 was used to filter out rRNA [67]. Then, the raw short reads were processed through in-house Perl scripts. In this step, clean reads and more reliable results were obtained by removing reads that contain adapter, low-quality reads with less than 50% bases with quality scores lower than 5 and unknown bases that were larger than 10% N bases. At the same time, GC-content, Q20, Q30 and sequence repetition level of clean data were calculated. Transcriptome de novo assembly was achieved using Trinity software with parameters of Kmer = 25 [68]. The short reads were assembled into contigs on the basis of their overlap regions. Then, CD-HIT (http://www.bioinformatics.org/cd-hit/) with 95% global sequence identity was used to obtain unigenes by clustering the sequences of the de novo assembled transcriptome to remove any redundancy.

### Functional annotation of gene transcripts

For functional annotation, the assembled unigenes were analyzed by BLAST to by using the Nucleotide database (Nt) with 10–5 E-value threshold [69]. The unigenes were annotated using the BLASTx tool with E-value < 10^− 5^ against the NCBI non-redundant protein sequences database (Nr), Swiss-Prot protein database, and eukaryotic Cluster of Orthologous Groups of proteins (KOG) database [69]. The search results were imported into Blast2GO version b2g4pipe_v2.5 for gene ontology (GO) assignments [70]. The KOBAS tool (Kegg Automatic Annotation Server) was used for KEGG Orthology and KEGG pathway assignments. In addition, the unigenes were used as query sequences for searching the Pfam (Protein family).

### Gene expression quantification and differential expression analysis

Gene expression quantification was performed using the Bowtie aligner and expectation–maximization method (RSEM) to obtain the number of read counts via the Perl script align_and_estimate_abundance.pl with –est_method RSEM from the Trinity protocol [71, 72]. The expression levels of unigenes were normalized and calculated as the values of fragments per kilobase of transcripts per million mapped fragments (FPKM) during the assembly and clustering process [73]. After standardizing the read count data with TMM, the *p*-values of edgeR package analyses were adjusted via Benjamini-Hochberg’s method to determine the false discovery rate (FDR) and identify DEGs [74]. Then, the *p*-value obtained from the test was corrected to get the *q*-value. The standard of differential gene expression screening is |log2(FoldChange)| > 1 and *q*-value < 0.05.

### GO function and KEGG pathway enrichment analyses

To study the biological significance of DEGs, the GO database was employed to exhibit GO enrichment analysis of DEGs during the different treatments of Giant Juncao using the GOseq (v1.22) software [75]. Parameter settings was that the corrected *p*- value was less than 0.05. The various metabolic pathways of DEGs were analyzed by using the KEGG database. The statistical enrichment of DEGs was tested using the KOBAS 2.0 web server and the corrected *p*-value < 0.05 was considered to be significantly enriched in KEGG [76].

### TF analysis of DEGs

All identified DEGs were blasted with PlantTFDB (Plant Transcription Factor Database) 4.0 (http://planttfdb.cbi.pku.edu.cn/), and the threshold was set as 1 × e^− 5^.

### Weighted gene co-expression network analysis

WGCNA was used to explore the relationship between genes and physiological indexes, as well as that between genes and genes. The genes with FPKM less than 0.5 were filtered out, and the remaining genes were input into WGCNA network construction (WGCNA v1.69 package in R) [77]. Pearson correlation matrix and network topology analysis were used to calculate the gene correlation and soft thresholding power, respectively. Then, the adjacency was converted to a topological overlap matrix. In standard WGCNA networks, power, minModuleSize, and mergeCutHeight value were set to 7, 30, and 0.25, respectively. The networks were visualized using Cytoscape v3.7.2.

### Quantitative RT-PCR validation

To validate the accuracy of the RNA-seq results, quantitative RT-PCR analysis was conducted on a CFX Connect qPCR detection system. Two bHLH transcription factors were randomly selected, and PgACT gene was used as a reference gene (the primers are shown in Table S4 as supplementary data). The 2^-∆∆CT^ method was used to calculate the relative expression levels of genes.

### Statistical analysis of physiological data

Statistical analysis of physiological data was conducted using SPSS20.0 (SPSS Inc., Chicago, IL, USA). The significance of differences among every treatment was tested by one-way ANOVA and Duncan’s multiple comparative analysis (*P* < 0.05).

## Supplementary Information

**Additional file 1: Table S1-S4**. **Table S1**: Overview of the sequencing. **Table S2**: Unigene information annotated in different databases. **Table S3**: Number of differential genes annotated by KEGG pathway. **Table S4**: The primers of two bHLH TF and *PgACT*.

**Additional file 2.** GO enrichment in DEGs when FDR<0.01.

**Additional file 3.** KEGG enrichment in DEGs at different drought stress and rehydration conditions.

**Additional file 4.** DEGs associated with photosynthesis at different drought stress and rehydration conditions.

**Additional file 5.** DEGs associated with plant hormone signal transduction pathway at different drought stress and rehydration condition.

**Additional file 6.** TF analysis results.

**Additional file 7.** Degree and log_2_(FoldChange) value of hub genes under different treatments in the four modules.

## Data Availability

The transcriptome sequences have been deposited in NCBI under BioProject ID: PRJNA632455 and the URL is https://www.ncbi.nlm.nih.gov/bioproject/PRJNA632455 . All data generated or analyzed during this study are included in this published article and its supplementary information files (Additional files 1, 2, 3, 4, 5).

## Abbreviations

DEGs: Differentially expressed genes
TF: Transcription factors
bHLH: Basic helix-loop-helix
NAC: NAM (no apical meristem), ATAF1/2 and CUC2 (cup-shaped cotyledon);
FAR1: Fatty acyl-CoA reductase
MYB: Myeloblastosis
bZIP: Basic leucine zipper
SOD: Superoxide dismutase
POD: Peroxidase
PRO: Proline
MDA: Malondialdehyde
Pn: Net photosynthetic rate
Tr: Transpiration rate
Gs: Stomatal conductance
WUE: Water use efficiency
WGCNA: Weighted gene co-expression network analysis
