# Supplementary material for: Transcriptome profiling reveals the effects of drought tolerance in Giant Juncao
Source: BMC Plant Biol. 2021 Jan 4;21:2. doi: 10.1186/s12870-020-02785-7 (PMC7780708; doi:10.1186/s12870-020-02785-7)
Supplement: Supplementary file 1 — Additional file 1: Table S1-S4. Table S1: Overview of the sequencing. Table S2: Unigene information annotated in different databases. Table S3: Number of differential genes annotated by KEGG pathway. Table S4: The primers of two bHLH TF and PgACT. [file 12870_2020_2785_MOESM1_ESM.docx]

**Table S1**. Overview of the sequencing

| Gategory |  | C0 |  |  | D1 |  |  | D2 |  |  | R1 |  |  | R2 |  |  | R3 |  | Total |
| --- | --- | --- | --- | --- | --- | --- | --- | --- | --- | --- | --- | --- | --- | --- | --- | --- | --- | --- | --- |
|  | C0a | C0b | C0c | D1a | D1b | D1c | D2a | D2b | D2c | R1a | R1b | R1c | R2a | R2b | R2c | R3a | R3b | R3c |  |
| Raw reads | 56668724 | 57707276 | 55781112 | 53732668 | 55665182 | 55767080 | 54745398 | 54710708 | 54657872 | 56668638 | 56304184 | 56509112 | 56668738 | 56668884 | 56665606 | 53223072 | 55372048 | 56666176 | 1004182478 |
| Clean reads | 54780044 | 55893312 | 53926936 | 51902178 | 53426076 | 53507940 | 52382982 | 52348504 | 52488416 | 54750744 | 54292602 | 54577292 | 54442162 | 54267986 | 54179800 | 51285000 | 53349718 | 54701804 | 966503496 |
| Cleanbases(Gb) | 8.22 | 8.38 | 8.08 | 7.78 | 8.02 | 8.02 | 7.86 | 7.86 | 7.88 | 8.22 | 8.14 | 8.18 | 8.16 | 8.14 | 8.12 | 7.7 | 8 | 8.2 | 144.96 |
| Q20 (%) | 98.11 | 97.81 | 97.97 | 98.00 | 98.02 | 98.04 | 97.81 | 97.61 | 97.87 | 97.18 | 97.79 | 97.82 | 97.81 | 97.78 | 98.00 | 97.92 | 97.80 | 97.92 |  |
| Q30 (%) | 94.78 | 94.06 | 94.45 | 94.53 | 94.59 | 94.60 | 94.20 | 93.69 | 94.29 | 92.65 | 94.04 | 94.13 | 94.11 | 94.04 | 94.44 | 94.29 | 94.08 | 94.37 |  |
| GC (%) | 56.21 | 56.58 | 56.35 | 56.28 | 55.67 | 56.22 | 55.83 | 55.73 | 55.98 | 56.22 | 56.03 | 56.29 | 57.65 | 57.17 | 57.35 | 56.97 | 57.76 | 57.72 |  |
| Error (%) | 0.02 | 0.025 | 0.025 | 0.02 | 0.02 | 0.025 | 0.025 | 0.025 | 0.025 | 0.025 | 0.025 | 0.025 | 0.025 | 0.025 | 0.025 | 0.025 | 0.025 | 0.025 |  |

The control (0 day) and drought treated (7 days, 14 days) and rehydration treated (1 day, 5days, 9 days) were generated and referred to as C0, D1, D2, R1, R2 and R3. Each condition had three biological repetition and those were referred to as a, b and c.

**Table S2**. Unigene information annotated in different databases

| Public Database | Number of Unigenes | Percentage (%) |
| --- | --- | --- |
| Annotated in Nr | 46952 | 50.0 |
| Annotated in Nt | 47268 | 50.3 |
| Annotated in KEGG | 14221 | 15.1 |
| Annotated in Swiss Prot | 30901 | 32.9 |
| Annotated in Pfam | 40136 | 42.7 |
| Annotated in GO | 26618 | 28.4 |
| Annotated in KOG | 24334 | 25.9 |
| Annotated in all Databases | 6963 | 7.4 |
| Annotated in at least one Database | 57941 | 61.7 |
| Total Unigenes | 93907 | 100 |

**Table S3**. Number of differential genes annotated by KEGG pathway

| Comparison | DEGs list | DEGs upregulated | DEGs downregulated |
| --- | --- | --- | --- |
| D1 | 4303 | 1972 | 2337 |
| D2 | 7093 | 4490 | 5725 |
| R1 | 4221 | 2166 | 2096 |
| R2 | 6000 | 3106 | 3229 |
| R3 | 3141 | 1126 | 2016 |

Compared with control (0 day), drought treated (7 days, 14 days) and rehydration treated (1 days, 5days, 9 days) were generated and referred to as D1, D2, R1, R2 and R3, respectively.

**Table S4**. Primer sequences used for qRT-PCR

| Gene ID | Sense Primer | Anti-sense Primer |
| --- | --- | --- |
| *c104644_g1_i1* | AAGGTGAAGGAACGGAAT | GTGGAGAACAAGGCTAATC |
| *c109045_g3_i1* | TACCATATCCGTCACAAC | CGAGAATCCACAATCAGA |
| *PgACT* | TGCTCAGTGGAGGGTCTACCAT | ACAACAGCCATCATCCAGAA |
